# Imaging of Clot by ^99m^Tc-HMPAO Labeled Platelet in Animal Model Induced Thrombosis

**DOI:** 10.22037/ijpr.2020.1101234

**Published:** 2020

**Authors:** Mahdieh Parvizi, Saeed Farzanefar, Abbas Tafakhori, Mahdi Gholami, Fariba Johari Daha, Hana Saffar, Ali Khalaj, Mehrshad Abbasi

**Affiliations:** a *Department of Radiopharmacy, Faculty of Pharmacy, Tehran University of Medical Sciences, Tehran, Iran. *; b *Department of Nuclear Medicine, Vali-Asr Hospital, Tehran University of Medical Sciences, Tehran, Iran. *; c *Department of Neurology, School of Medicine, Imam Khomeini Hospital, Tehran University of Medical Sciences, Tehran, Iran. *; d *Department of Toxicology and Pharmacology, Faculty of Pharmacy, Tehran University of Medical Sciences, Tehran, Iran. *; e *Radiation Application Research School, Nuclear Science and Technology Research Institute, NSTRI, Tehran, Iran. *; f *Department of Pathology, Cancer Institute, Imam Khomeini Hospital, Tehran University of Medical Sciences, Tehran, Iran.*; g *Department of Medicinal Chemistry, Faculty of Pharmacy, Tehran University of Medical Sciences, Tehran, Iran.*

**Keywords:** ^99m^Tc-HMPAO, Labeled platelet, Thrombosis, Animal model, Scintigraphy

## Abstract

^99m^Tc-HMPAO labeled platelet (LP) imaging may integrate thrombosis imaging into routine clinical procedures. In the current study, we assessed the feasibility of the use of ^99m^Tc-HMPAO LP for imaging of small clots in an animal model. Thrombosis was induced by application of FeCl_3_ solution in the distal part of the inferior vena cava (IVC) of a 6100 g anesthetized rabbit and in a male Wistar rat weighing 420 g. Twenty minutes later, 178 MBq ^99m^Tc-HMPAO LP was injected. ^99m^Tc-HMPAO LP preparation was done as defined and standardized in a previous report. Whole body and SPECT imaging were done 60, 90, and 120 min after tracer injection. Then, the clotted part of the vein was extracted and then its activity and pathologic evaluations were compared with the proximal part of the IVC at a similar volume. A 17 × 6 mm clot was clearly detected with both planar and SPECT imaging. The count to pixel ratio (CPR) of the clotted part of the vein was 35, 40, and 40 compared to the non-clotted vein (*i.e.* 19, 18, and 21) at 60, 90, and 120 min, respectively. After clot extraction, the CPR decreased to 14. The clot activity was 0.44 MBq compared to 0.01 MBq of the normal control vein. Also, clot induction was pathologically proven. ^99m^Tc-HMPAO LP preparation is logistically possible in clinical nuclear medicine and the ability of imaging small size clots encourages future trials for real clinical thrombotic scenarios.

## Introduction

Thrombosis and thromboembolism are etiology of certain significant diseases including ischemia of the extremities, the myocardium, and intestine as well as pulmonary thromboembolism and cerebral venous sinus thrombosis (1). There are several imaging techniques to detect thrombosis. These methods include anatomical imaging with CT and MRI which delineate the clot in the vessel or ultrasonography which shows the flow of the vessel and its compressibility (2-4). Ultrasonography is promising with the use of advent methods employing microbubble or nanoparticle. Nevertheless, detection of deep venous thrombosis in the abdomen and pelvis as well as in the cerebral veins located in the skull is not possible clinically by the ultrasonography (5). CT scan and CT angiography illustrate the clot within the veins but diagnostic accuracy is not generally acceptable. MRI and MR venography are used to detect the cerebral sinus thrombosis with frequent suboptimal diagnostic performance (6).

In nuclear medicine, macroaggregated albumin (MAA) has also been used to detect pulmonary thromboembolism (7). Furthermore, antibodies and peptides have been examined and proved successful for detection of platelet aggregation in certain thrombotic conditions (8, 9). ^99m^Tc-HMPAO labeled platelets were first developed in 1988, but there have been few reports on its application ever since (10-12). Extraction of the platelets is time consuming and rather hard hence precluded clinical use of labeled platelets (13). To best of our knowledge, the human biodistribution of labeled platelets, required activity for preclinical and also clinical imaging and optimal imaging timing for clot are not yet reported. Moreover, the capability of the clinical imaging of labeled platelets for detection of small clots is not well documented. We recently reported the bio-distribution of ^99m^Tc-HMPAO in rabbits and mice. We also reported the preparation of ^99m^Tc-HMPAO labeled platelets was not easy but not too difficult to hinder its clinical use (14). ^99m^Tc-HMPAO is not stable and needs quality control and ongoing replacement of suboptimal kits. There is subtle evidence about the practical use of this scan in literature, so we evaluated its feasibility and performance in clinical practice in the current study. Moreover, we assessed the capability of labeled platelets to image the clot by clinically available gamma cameras.

## Experimental

The specifications of the instruments and tools used in this study were summarized in our previous paper (14). The sterility of all material and instruments including the syringes and tubes was assured by examining the integrity of the sealed coverage with regards to the good laboratory practice standards.

*Labeling*

Labeled platelets were prepared according to our previous report (14). In brief, the platelets were extracted from the whole blood by two-step centrifugation at 400 and 1050 G for 15 min. The platelets, sedimented at the bottom of tube as a platelet pellet, were suspended in acid citrate dextrose (ACD) and saline (1 mL: 6 mL) for pH adjustment. The supernatant was stored as platelet poor plasma (PPP) at 37 °C for future use. The extracted platelets were labeled with 1332 MBq ^99m^Tc-HMPAO in 2 kits. HMPAO is produced and distributed by Pars Isotope Co (Tehran, Iran). The kits contain 0.5 mg HMPAO (both D and L isomers), 7.6 µg stannous chloride dihydrate, and 4.5 mg sodium chloride, and are sealed under nitrogen atmosphere. For ^99m^Tc-HMPAO preparation, ^99m^Tc-pertechnetate fresh elution of ^99^Mo/^99m^Tc generator (Pars Isotope Co, Tehran, Iran) was added to the HMPAO kit and shook for 1 to 2 min (15). ^99m^Tc-HMPAO was added dropwise into the platelet suspension and incubated at 37 °C for 25 min. Afterward, 3 mL PPP was added to the solution and centrifuged for the 3^rd^ time at 500 G for 10 min to separate unbound ^99m^Tc-HMPAO from labeled platelets. The supernatant including unbound ^99m^Tc was removed and the ^99m^Tc-HMPAO labeled platelets were collected at the bottom of the tube. Labeling efficiency was evaluated by counting the yellowish supernatant and creamy platelet pellet in about 2 mL of the sediment at the bottom of the tube. The labeled platelets were re-suspended in 5 mL PPP and reinjected into the rabbit. The procedure could be done with 50 mL whole blood and 740 to 1110 MBq ^99m^Tc for each kit for which blood samples were drawn from two rabbits via heart puncture. Quality control tests were done according to our previous report (14). In brief, TLC assay was done for HMPAO radiochemical purity. Viability of eosin dye exclusion test, stability in human serum, the ratio of release of ^99m^Tc-labeled out of the platelets, and labeling efficiency were done. For HMPAO kit, pyrogenicity with Limulus amebocyte lysate (LAL) kit and sterility tests in soybean digest medium, and thioglycollate medium were also employed (15-18).

*Animal handling*

A male Wistar rat weighing 420 g was used to induce the clot in the inferior vena cava (IVC) via a transverse laparotomy line (19, 20). We tried the procedure on the rat before proceeding to repeat it in the rabbit to make sure of the success of clot induction. Two female New Zealand White rabbits weighing about 3800 g and 5500 g were used for blood donation and another female New Zealand White rabbit weighing 6100 g was used for thrombosis induction, labeled platelet injection, and imaging (21). The rabbits were transferred from Pasteur Institute of Iran and Faculty of Pharmacy, Tehran University of Medical Sciences via a maximum 1-hour drive into the Nuclear Medicine Department (Vali-Asr Hospital, Tehran University of Medical Sciences). The rabbits were anesthetized with ketamine 10% (70 mg/kg) and xylazine 2% (10 mg/kg) prior to the procedure. Through heart puncture, 38 and 29 mL blood samples were drawn in 50 and 20 mL syringes containing ACD solution (Pars Isotope Co, Tehran, Iran). The total volume of the blood and ACD summed up to 85 mL.

*Thrombosis induction*

Ferric chloride (FeCl3, Loba Chemie, Mumbai, India) was dissolved in distilled water to acquire 20% (w/v) ferric chloride solution. Two milliliter of the solution was filtered by a 0.20 µ sterile filter (CA-membrane, LOT NO: 16534). Whatman No.1 paper was cut at 2 × 0.5 cm for the rat and 2.5 × 1.5 cm for the rabbit and soaked in the ferric chloride solution for 10 min (22). After exposure and successful isolation of IVC, the paper was wrapped around the exposed IVC in the rat and rabbit. The paper was kept wet by one or two droplets (50 to 100 µL) of the ferric chloride solution twice until the color of the vein changed from pink to brown. This change usually occurred 15 min after placing the paper (23). In the rat, the clotted part and a nearby normal part of the IVC was sent for pathologic evaluation. The rabbit was directly re-injected by 178 MBq ^99m^Tc-HMPAO-platelets at a volume of 5 mL via the marginal ear vein.

*Pathologic examination*

For determination of successful clot induction in the rat and rabbit and platelet extraction, the clotted and normal vein in formalin and smears and liquid sample of the platelet pellet re-suspended in normal saline were sent to Department of Pathology (24). Direct smears were stained using the Wright and Giemsa method. The liquid material was cytocentrifuged at 500 g for 10 minutes and subsequently stained by the Wright and Giemsa method. Moreover, two fragments of formalin fixed tan-gray tubular soft tissue were submitted, one labeled as a normal vessel and the other as an induced thrombosis in the rat and rabbit model. The specimens were processed and stained with Hematoxylin and Eosin.

*Imaging*

Twenty minutes after induction of the thrombosis and suturing of the transverse laparotomy incision in one layer, the rabbit was injected with 178 MBq ^99m^Tc-HMPAO labeled platelets. Injection was done directly into the marginal ear vein while anesthesia was maintained by hourly ketamine-xylazine injections. Imaging started 60 min after tracerinjection. Anterior and posterior planar static imaging of 1500 and 1000 k counts was done for about 2.5-3 min by a dual head gamma camera (AnyScan, Mediso, Budapest, Hungary) in the Department of Nuclear Medicine, Vali-Asr Hospital (TUMS, Tehran, Iran). Then, SPECT acquisition was done with 15 second projection time, azimuth of 5.6 and matrix size of 128 × 128. The SPECT image was reconstructed using the MOSEM iterative method. Then, the rabbit was killed by intravenous ketamine and xylazine overdose, and the clotted part of the vein was surgically removed. The length of the clotted vein was 17 mm that was stored in a total volume of 5 mL formalin for pathologic examination. The control proximal part of the vein at a rather similar volume was removed and stored in the same condition for pathologic examination. The activity of the clotted vein and the normal vein was measured by several counting tries in a dose calibrator (CRC®15R, Capintec, Ramsey, New Jersey). After sacrificing the rabbit and removing the clot containing vein, another 1000 K count image was acquired. The images were inspected by a nuclear physician blinded to the procedure, but aware that a clot was induced somewhere in the rabbit. The region of interest (ROI) count was calculated on the clot-containing and normal IVC, liver, spleen, left kidney, and bladder.

## Results

**Figure 1:**
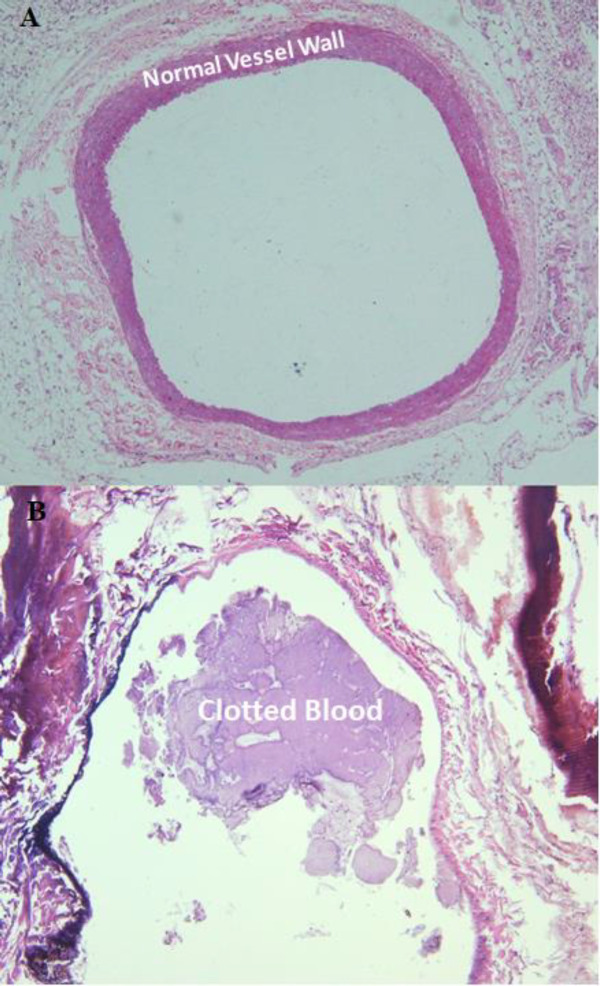
(A) Pathological examination of a cross section of the normal vein and (B) induced thrombosis in the rabbit’s IVC; thrombosis was induced by application of filtered FeCl_3_ solution stained Whatman No. 1 paper around the dissected IVC *in-vivo*

**Figure 2:**
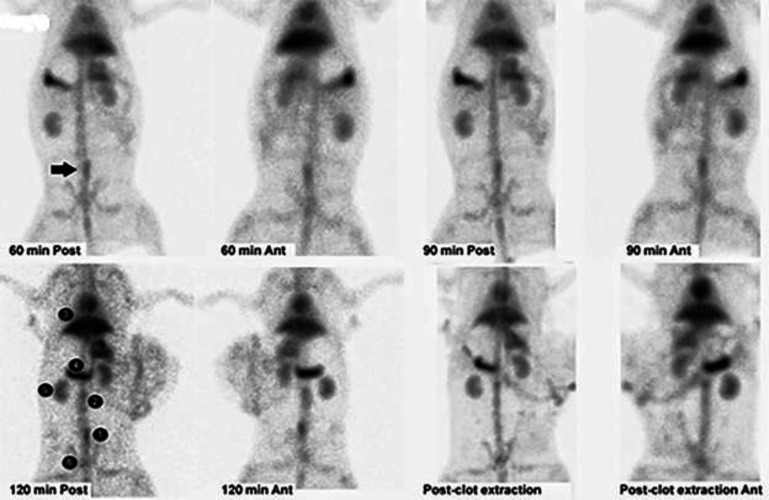
Planar images 60, 90, and 120 min after 178 MBq ^99m^Tc-HMPAO labeled platelet injection into the rabbit with thrombosis in the IVC (black arrow) in the anterior and posterior images. Images after clotted vein extraction are also shown. Quantification of the activity within the region of interests around the clot [1], blood pool of the inferior vena cava [2], liver [3], spleen [4], left kidney [5] and bladder [6]

**Figure 3:**
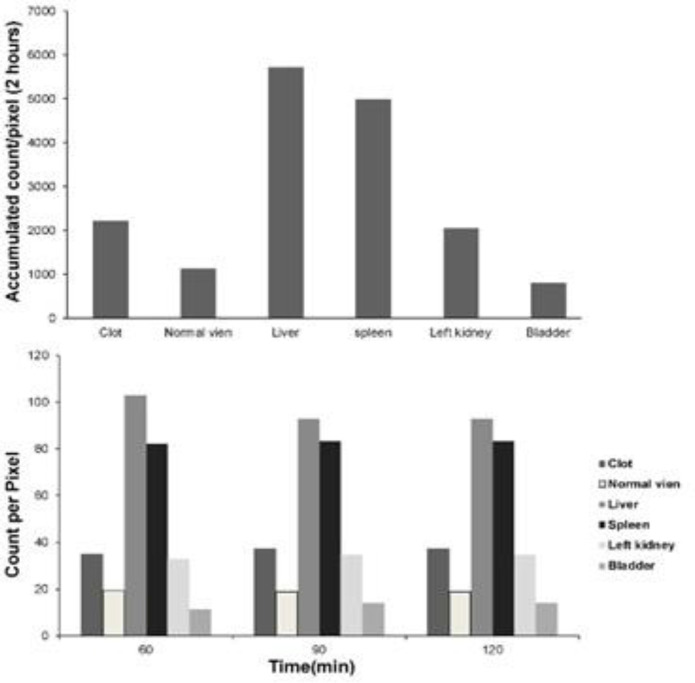
Accumulative dose and the quantification of 60, 90, and 120 min images after 178 MBq ^99m^Tc-HMPAO labeled platelet injection into the rabbit with thrombosis in the IVC

**Figure 4:**
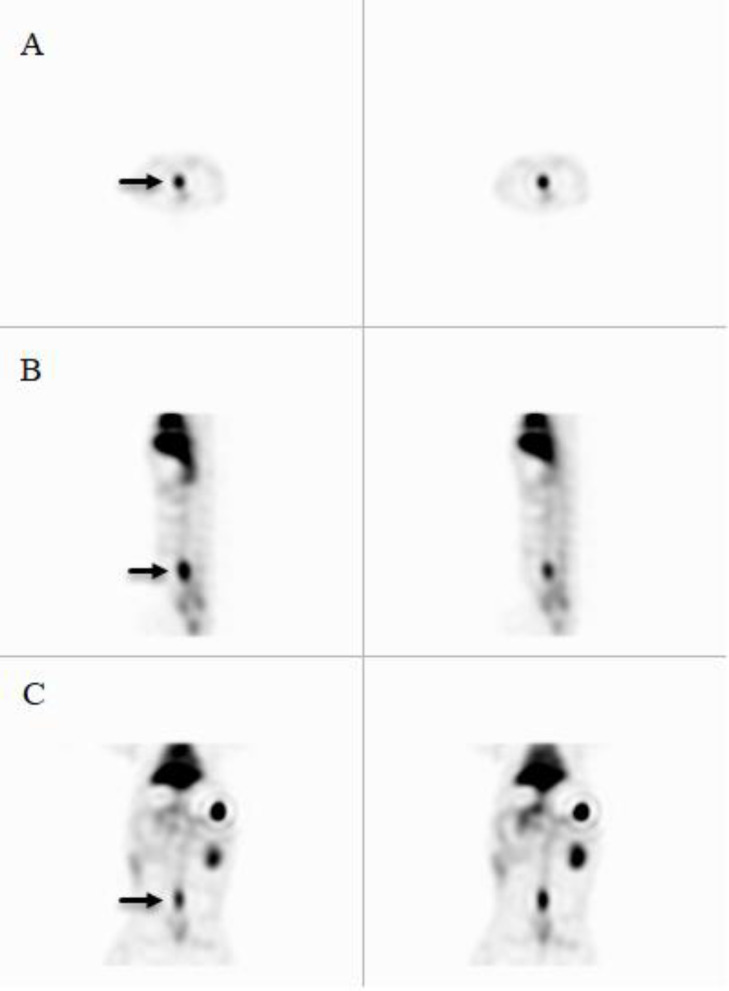
SPECT images 65 min after injection of 178 MBq ^99m^Tc-HMPAO labeled platelets into the rabbit with thrombosis (black arrow) in the IVC. (A) Transverse, (B) sagittal, and (C) coronal

Quality control parameters for the HMPAO kit, isolated platelets, and labeled platelets were within the acceptable range according to our previous reports (14). On microscopic examination of both direct and cytocentrifuged smears of the specimen supposed to be platelets, a large number of platelets were identified with a rare scattered population of lymphocytes in the background (*i.e.* 3-4 in high power field). The IVC was successfully exposed to induce thrombosis. Pathologic examination of the rat and rabbit veins indicated successful thrombosis induction (Figure 1). Both 1000 k count and 1500 K count planar and SPECT images were interpretable. The images illustrated uptake in the liver, spleen, and kidneys. The uptake in the thyroid and bladder was minimal at the time of imaging. The blood pool of the main vessels, heart, and bone/bone marrow was noticeable (Figure 2). A high activity was determined in the inlet of the pelvis more defined on the SPECT image as a cylindrical abnormality anterior to the vertebral column in the line of blood pool of the IVC (Figure 4). The count per pixel of the ROIs over these organs is presented in Figure 2. The count/pixel was 34.4, 39.6, 39.5 for the clotted vein and 19.6, 18.1, 25.0 for the normal vein at 60, 90, and 120 min, respectively. The area under the curve in the ROIs during 2 h after injection was 1878.6, 987.1, 4709.4, 4108.9, 1804.0, and 777.7 count/min/pixel for the clotted vein, normal vein, liver, spleen, kidney, and bladder, respectively (Figure 3). The post scarification image after extraction of the clotted vein indicated removal of denoted abnormality and reduced count/pixel to 14 (Figure 2). The ratio of the count per pixel of the vein to the liver was 0.3, 0.4, and 0.5 for clotted vein at 60, 90, and 120 min, respectively, and 0.3 after extraction of the clotted vein. The diameter of the clot was about 6 mm and it elongated along the vein for 17 mm. The mean activity of the clotted vein was 0.44 MBq. The activity of the control vein was zero.

## Discussion

We demonstrated that a 6 mm clot was imaged by clinical gamma cameras after injection of ^99m^Tc-HMPAO labeled platelets. The best imaging time was 90 and 120 min after tracer injection when the clot to liver uptake was optimal. This scan may be used for detection of any suspicious thrombotic condition in the human body (25, 26). The most challenging clinical question is pulmonary thromboembolism and deep vein thrombosis (27, 28), with clots in the pulmonary arteries which are larger than the clot imaged in the current study (29). The segmental pulmonary arteries are larger than 3 mm and the surrounded clot may be successfully imaged by this method (30). The lung parenchyma provides a very low background uptake for optimal hot imaging of the platelet labeled clots. Furthermore, the scan could be evaluated for less frequent conditions including pseudotumor cerebri with possible etiology of venous thrombosis, a topic which is currently under investigation in our department (31). Since the platelets do not pass the blood brain barrier, the background of the scans should be theoretically low enhancing the diagnostic efficiency for detection of intracranial thromboses (32). We showed that the procedure for preparation of ^99m^Tc-HMPAO labeled platelets was practical in academic nuclear medicine centers. The injection of platelets is concerning, particularly, if the platelets become stimulated by the labeling procedure and can exacerbate thrombosis upon delivery; control studies should be conducted before proceeding to the clinical phase to assure the safety of the scan.

There are many approaches for detection of the thrombosis including Doppler ultrasonography, CT angiography, MR angiography, perfusion imaging with MAA, and clot detection with specific anti-platelet antigen antibodies (9, 33-36). Although angiography and venography via catheterization are the standard methods in many conditions, they are invasive and less clinically indicated. There are limitations for any currently available thrombus imaging method: extremity or calf ultrasonography is not accurate for pelvic veins, the source of the majority of pulmonary thromboembolisms. CT angiography may cause contrast-induced nephropathy and has limited accuracy for detection of thrombosis in cerebral veins or in the particular conditions including post-transplant patients (37). MRI and MR angiography offer a high contrast for detection of clots (38); nevertheless, certain thrombosis, including cerebral vein thrombosis particularly chronic cases, are essentially less possibly detectable by anatomical imaging modalities, including MRI (39). Among nuclear medicine methods, imaging by MAA, a cold spot imaging has naturally low contrast and many antibodies based tracers, unfortunately, provoke autoimmune process. Labeled fibrin imaging is applicable in the acute phase of thrombosis and the accuracy of the ^99m^Tc-apcitide scan is low for pulmonary thromboembolism (40, 41). Platelet imaging may fit within the gap of these imaging techniques and promotes thrombosis imaging. Furthermore, the etiology study of many conditions including transplantation rejection could be further enhanced by the use of labeled platelets.

Relative clot tracer accumulation was optimal compared to the liver and spleen which are the target organs for platelets. The contrast of the image was acceptable at the time of imaging (*i.e.* 60 min after injection) with enhancement after 90 to 120 min. The short imaging time for ^99m^Tc-HMPAO labeled platelets scan is remarkably more applicable than delayed imaging time for ^111^In platelet scan which is a drawback for patients in need of prompt diagnosis (42). The tracer was injected 20 min after clot induction. It is not clear to us that had the tracer been injected later, the imaging of the clot would have been possible. In other words, it should be tested to what extend chronic thrombosis or even thromboembolic clots could absorb circulating platelets making it possible to image chronic clots with labeled platelets. There are certain reports in favor of the idea that thrombosis is a dynamic process and circulating platelets may involve the structure of the clot (43). Future studies should evaluate the duration platelets remain in the animal and when blood clearance occurs. In the current study, the site of activity corresponded to the clot and no activity was detected in the distal part where a secondary propagation of the clot is expected distal to the induced clot site. This finding fosters the idea that injected labeled platelets could replace clot platelets; hence, the scan might be applicable in chronic (*i.e.* 24 h) thrombotic conditions.

FeCl_3_ solution induces thrombosis via endothelium layer destruction and enhancement of platelet attachment to collagen, fibrinogen, and Von Willebrand factor (23). The vein was microscopically examined to assure that the vein contained the clot and necrosis was not the predominant reason for vein flow closure. Deposition of the tracer within the clot was proved not only by imaging but also by activity measurement of the surgically removed thrombotic vein compared to the normal vein.

## Conclusion

We documented that ^99m^Tc-HMPAO labeled platelets could detect a small size clot using a clinically available gamma camera. We detected a 6 mm clot through injection of 178 MBq ^99m^Tc-HMPAO tagged platelets. The method we employed is easily applicable with 3 rounds of centrifugation in ordinary nuclear medicine centers. Nevertheless, the study was small, as it was conducted in one rabbit, and additional clarifications and experiments are needed to provide additional information on the method to ensure the study is of significant value to the scientific research community. Human studies should be designed for further evaluation of the bio-distribution of autologous platelets in humans and the diagnostic accuracy of the scan.

## Custom

All applicable institutional and/or national guidelines for the care and use of animals were followed.

## Availability of data and material

The datasets used and analyzed during the current study are available from the corresponding author on reasonable request.
